# Improvement of thermostability and catalytic efficiency of glucoamylase from *Talaromyces leycettanus* JCM12802 via site-directed mutagenesis to enhance industrial saccharification applications

**DOI:** 10.1186/s13068-021-02052-3

**Published:** 2021-10-16

**Authors:** Lige Tong, Jie Zheng, Xiao Wang, Xiaolu Wang, Huoqing Huang, Haomeng Yang, Tao Tu, Yuan Wang, Yingguo Bai, Bin Yao, Huiying Luo, Xing Qin

**Affiliations:** State Key Laboratory of Animal Nutrition, Institute of Animal Sciences, Chinese Academy of Agricultural Sciences, Beijing, 100193 China

**Keywords:** Glucoamylase, Thermostability, Catalytic efficiency, Site-directed mutagenesis, Industrial application

## Abstract

**Background:**

Glucoamylase is an important industrial enzyme in the saccharification of starch into glucose. However, its poor thermostability and low catalytic efficiency limit its industrial saccharification applications. Therefore, improving these properties of glucoamylase is of great significance for saccharification in the starch industry.

**Results:**

In this study, a novel glucoamylase-encoding gene *TlGa15B* from the thermophilic fungus *Talaromyces leycettanus* JCM12802 was cloned and expressed in *Pichia pastoris*. The optimal temperature and pH of recombinant *Tl*Ga15B were 65 ℃ and 4.5, respectively. *Tl*Ga15B exhibited excellent thermostability at 60 ℃. To further improve thermostability without losing catalytic efficiency, *Tl*Ga15B-GA1 and *Tl*Ga15B-GA2 were designed by introducing disulfide bonds and optimizing residual charge–charge interactions in a region distant from the catalytic center. Compared with *Tl*Ga15B, mutants showed improved optimal temperature, melting temperature, specific activity, and catalytic efficiency. The mechanism underlying these improvements was elucidated through molecular dynamics simulation and dynamics cross-correlation matrices analysis. Besides, the performance of *Tl*Ga15B-GA2 was the same as that of the commercial glucoamylase during saccharification.

**Conclusions:**

We provide an effective strategy to simultaneously improve both thermostability and catalytic efficiency of glucoamylase. The excellent thermostability and high catalytic efficiency of *Tl*Ga15B-GA2 make it a good candidate for industrial saccharification applications.

**Supplementary Information:**

The online version contains supplementary material available at 10.1186/s13068-021-02052-3.

## Background

Glucoamylase (1,4-α-d-glucan glucohydrolase, EC 3.2.1.3) is one of the most important and widely used industrial enzymes, exhibiting great application potential in sugar, ethanol, bread, beer, textile, and pharmaceutical industries [1]. Glucoamylase could catalyze the hydrolysis of α-1,4 and α-1,6 glycosidic linkages in starch and related oligosaccharides from non-reducing ends to release β-d-glucose [2]. Currently, commercial glucoamylases are mainly obtained from filamentous fungi, such as *Aspergillus niger*, *Rhizopus niveus,* and *R. delemar*, displaying moderate thermostability and slow catalytic activities [3, 4]. However, the industrial conversion of starch to glucose consists of a liquefaction process at 95–105 ℃ for 2 h and a saccharification process at 60 ℃ for 48–72 h. Switching from the liquefaction to the saccharification process requires additional specialized equipment for cooling to ensure the catalytic integrity of glucoamylase [5]. The poor thermostability of current glucoamylases results in great energy consumption and process inconvenience to undergo the saccharification process in starch industries.

Generally speaking, enzyme discovery and engineering are the major approaches for acquiring enzymes with higher thermostability and catalytic efficiency. Moreover, the search for thermostable glucoamylases derived from thermophilic microorganisms has attracted immense attention over recent years. So far, several thermostable glucoamylases have been identified from *A. wentii*, *A. oryzae*, *A. flavus*, *Fomitopsis palustris*, *Chaetomium thermophilum*, *Rhizomucor pusillus*, *Thermoanaerobacter tengcongensis,* and *Sulfolobus solfataricus*, all of which exhibit optimal temperature or thermostability above 60 ℃ [3, 6–12]. Nevertheless, further research is needed pertaining to the industrial aspect of the saccharification process. Reconstructing enzymes by protein engineering is another efficient way to obtain glucoamylases with high thermostability. Based on previous research, directed evolution and rational design, such as in the construction of disulfide bonds, result in significant thermostability improvement of glucoamylases. [13–16]. However, due to the activity-stability trade-off, an increase in enzyme thermostability is accompanied by a decrease in catalytic efficiency [17, 18].

Recently, it has been reported that increasing the overall rigidity of protein while minimizing influence on the active site could render improved thermostability either without affecting catalytic activity or ideally with positively promoting catalytic activity [19]. Moreover, many previous studies on phytase, β-glucanase, and xylanase thermostability engineering support this conclusion [20–22]. Such studies indicate that selecting mutant sites in a region distant from the catalytic center may be feasible to design enzymes with increased thermostability and catalytic efficiency.

*Talaromyces leycettanus* JCM12802 is a typical thermophilic fungus with an optimum temperature for growth at 40 ℃, producing a wide range of thermostable glycoside hydrolases, such as cellulase, β-glucosidase, and mannanase [23–25]. In this study, a novel thermostable glucoamylase *Tl*Ga15B from *T. leycettanus* JCM12802 was expressed and characterized. In order to further improve thermostability, *Tl*Ga15B mutants were rationally designed by introducing disulfide bonds and optimizing residual charge–charge interactions in a region distant from the catalytic center. Moreover, the application potential of the best mutant was evaluated by comparison with a commercial glucoamylase commonly used in the starch industry for saccharification.

## Results and discussion

### Cloning and sequence analysis of *Tl*Ga15B

In the present study, a novel glucoamylase-encoding gene *TlGa15B* was identified in the genome of thermophilic fungus *T. leycettanus* JCM12802, and the corresponding cDNA sequences were successfully obtained from the fungus grown in PDB medium. The *TlGa15B* was interrupted by 4 introns, resulting in an open reading frame of 1842 bp. Deduced *Tl*Ga15B contained 614 amino acid residues and harbored a signal peptide of 20 residues based on predictions established by the SignalP 5.0 program [26]. The isoelectric point and calculated molecular weight of glucoamylase *Tl*Ga15B were 4.76 and 63.3 kDa, respectively. *Tl*Ga15B shared the highest identity (69.85%) with a previously reported glucoamylase derived from *Rasamsonia emersonii* [27]. The sequence and structure analysis showed that *Tl*Ga15B was a typical glucoamylase, containing a catalytic domain of GH15 and a carbohydrate-binding domain of CBM20. Glu211, and Glu433 was predicted to be the catalytic residues that served as the proton donor and acceptor, respectively. Moreover, five highly conserved residues associated with substrate binding in the GH15 family were identified in the catalytic domain of *Tl*Ga15B, including Arg86, Asp87, Leu209, Trp210, Glu212, and Arg338 [28]. In addition, the modeled structure of *Tl*Ga15B was constructed using the glucoamylase from *Penicillium oxalicum* (PDB: 6FHV, 58% sequence identity and 94% coverage) as template with the help of SWISS-MODEL. Meanwhile, the Ramachandran plot was used to evaluate the quality of the structural model. There were only two amino acid residues observed in disallowed region (Additional file 1), indicating that the modeled structure of *Tl*Ga15B was credible.

### Expression and purification of recombinant *Tl*Ga15B

*Pichia pastoris*, the microbial expression system most widely used for the large-scale production of commercial enzymes, was used as the recombinant expression host to express glucoamylase *Tl*Ga15B [29]. The cDNA fragment coding for the mature *Tl*Ga15B was inserted into PIC9 and successfully expressed in *Pichia pastoris* GS115. After methanol induction, the glucoamylase activity of the culture supernatant reached 5000 U/L at 48 h. After purification by anion exchange, the recombinant *Tl*Ga15B had an apparent molecular weight of ~65 kDa on the SDS-PAGE (Additional file 2), which was close to the theoretical molecular weight.

### Biochemical characterization of purified recombinant *Tl*Ga15B

**Fig. 1:**
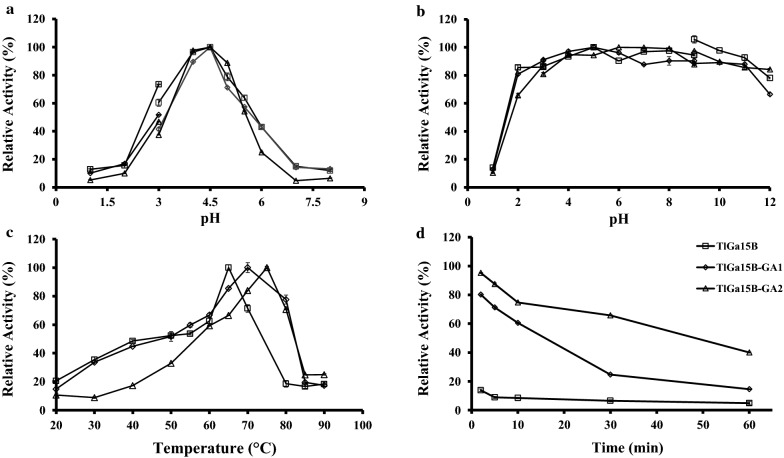
Effect of pH and temperature on the activity and stability of purified recombinant *Tl*Ga15B and mutants *Tl*Ga15B-GA1 and *Tl*Ga15B-GA2. **a** Effect of pH on the activity at 37 ℃. **b** Effect of pH on the stability at 37 ℃. **c** Effect of temperature (20–90 ℃) on the activity. **d** Effect of temperature (75 ℃) on the stability

The optimal pH of purified recombinant *Tl*Ga15B was pH 4.5 (Fig. 1a). Similar pH optima for glucoamylase activities had been reported from *C. thermophilum* pH 4.5–5.0 [11], *A. tritici* pH 4.0–5.0 [30], and *Bispora* sp. 4.0 [31]. Meanwhile, *Tl*Ga15B exhibited high stability in pH values ranging from 2.0 to 12.0 (Fig. 1b). The residual hydrolytic activity of *Tl*Ga15B after 1 h of incubation was over 75% of the original activity prior to incubation. These results suggested that *Tl*Ga15B was an acidic glucoamylase with broad-range pH stability.

As shown in Fig. 1c, the hydrolytic activity of purified recombinant *Tl*Ga15B increased with increasing temperature, reaching a maximum at 65 ℃. The optimal temperature was the same as the most widely used glucoamylases from *A. niger* [1, 32]. In terms of thermostability, *Tl*Ga15B from *T. leycettanus* JCM12802 exhibited excellent thermostability. *Tl*Ga15B retained 79.5%, 54.8%, and 36.7% of hydrolytic activity after 1 h of incubation at 55 ℃, 60 ℃, and 65 ℃, respectively (Additional file 3). When the temperature increased to above 75 ℃, the thermostability of *Tl*Ga15B significantly decreased (Fig. 1d). In contrast, the thermostability of *Tl*Ga15B is superior to that of most reported glucoamylase, which are stable at temperatures below 55 ℃ [1].

In addition, the kinetic parameters of purified *Tl*Ga15B with respect to soluble starch were determined at 55 ℃. The *K*_m_ and *V*_max_ values of purified recombinant *Tl*Ga15B were 0.77 mg/mL and 719.2 μmol/min/mg, respectively (Table 1). Meanwhile, the specific enzyme activities of purified recombinant *Tl*Ga15B were also evaluated using various substrates, including soluble starch, amylose, glycogen, α-cyclodextrin, β-cyclodextrin, and γ-cyclodextrin. The results revealed that *Tl*Ga15B was active toward soluble starch, amylose, and glycogen, but not other substrates, such as α-cyclodextrin, β-cyclodextrin, and γ-cyclodextrin. The specific activity toward glycogen was higher than toward soluble starch and amylose. The specific activities of the purified recombinant *Tl*Ga15B to soluble starch, amylose, and glycogen were 496.2, 221.1, and 658.3 U/mg, respectively.

**Table 1:** The kinetic parameters of *Tl*Ga15B and mutants on soluble starch at 55 ℃

Enzyme	Specific activity (U/mg)	*K*_m_ (mg/mL)	*V*_max_ (μmol/min/mg)	*k*_cat_/*K*_m_ (mL/s/mg)
*Tl*Ga15B	496.2 ± 1.3	0.77 ± 0.08	719.2 ± 19.1	982.3
*Tl*Ga15B-GA1	805.8 ± 5.7	0.62 ± 0.07	900.9 ± 23.2	1529.0
*Tl*Ga15B-GA2	1054.5 ± 2.2	0.29 ± 0.02	1093.0 ± 22.0	3982.6

### Selection of the mutagenesis sites in *Tl*Ga15B

**Fig. 2:**
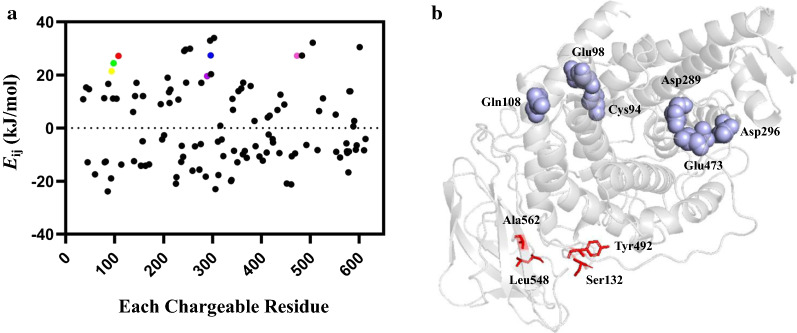
Total interaction energy of each chargeable residue in *Tl*Ga15B by ETSS analysis (**a**). The modeled structure of *Tl*Ga15B (**b**). Six candidate mutated sites away from the catalytic center were indicated in blue balls. The sites for introduction of disulfide bonds were shown as red sticks

It was reported that the construction of disulfide bonds and the optimization of residual charge–charge interactions were efficient strategies to improve the thermostability of industrial enzymes [33]. In this study, these protein engineering approaches were applied to enhance the thermostability of *Tl*Ga15B. Based on multiple sequence alignment and structure analysis, S132, Y492, L548, and A562 were chosen as targets to construct two disulfide bonds. The mutant *Tl*Ga15B-GA1 (S132C-Y492C/L548C-A562C) was constructed via PCR-mediated site-directed mutagenesis. As shown in Additional file 4, two disulfide-linked peptides SNPSGGLCT/SASGPCA (132C/492C) and PLWYCIV/SAIPCSA (548C/562C) were observed in the LC–MS/MS analysis, indicating that *Tl*Ga15B mutants formed disulfide bonds in the target sites. In addition, the enzyme thermal stability system (ETSS) was used to calculate the total interaction energy (*E*_*ij*_) between charged amino acids of *Tl*Ga15B [34]. 6 non-conserved amino acid residues (C94/E98/Q108/D289 /D296/E473) in a region distant from the catalytic center but in the catalytic domain of glucoamylase were selected for further analysis by ETSS (Fig. 2; Additional file 5). On account of the ETSS analysis, Q108 (total *E*_*ij*_ = 27.3 kJ/mol) was mutated to a neutral amino acid alanine (total *E*_*ij*_ = 2.3 kJ/mol), exhibiting the most significant decline in the *E*_*ij*_ value. Subsequently, the mutant *Tl*Ga15B-GA2 (S132C-Y492C/L548C-A562C/Q108E) was generated via site-directed mutagenesis using *Tl*Ga15B-GA2 as the template.

### Comparison of the enzymatic properties of *Tl*Ga15B and mutants

**Fig. 3:**
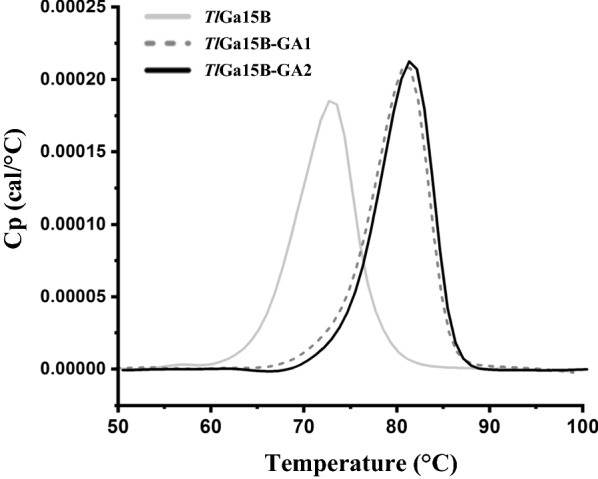
Thermograms of *Tl*Ga15B and mutants *Tl*Ga15B-GA1 and *Tl*Ga15B-GA2 detected using the DSC

As shown in Fig. 1a, b, there were no significant differences in pH optima and stability between *Tl*Ga15B and its mutants *Tl*Ga15B-GA1 and *Tl*Ga15B-GA2. By comparison, the optimal temperature and thermostability of mutants both significantly improved. The optimal temperatures of *Tl*Ga15B-GA1 and *Tl*Ga15B-GA2 were 70 ℃ and 75 ℃, which were 5 ℃ and 10 ℃ higher than that of *Tl*Ga15B, respectively (Fig. 1c). The *Tl*Ga15B incubated at 75 ℃ for 2 min only had 13.8% residual enzyme activity, while the mutant *Tl*Ga15B-GA1 still contained 60.6% and 24.6% of enzyme activity after incubation at 75 ℃ for 10 min and 30 min, respectively. Furthermore, the mutant *Tl*Ga15B-GA2 was extremely stable at 75 ℃, and 40% of enzyme activity remained after 1 h of incubation (Fig. 1d). Meanwhile, the DSC results supported the conclusion that both *Tl*Ga15B-GA1 and *Tl*Ga15B-GA2 exhibited higher thermostability than *Tl*Ga15B (Fig. 3). Compared with the *T*_m_ value of *Tl*Ga15B (72 ℃), the *T*_m_ values of *Tl*Ga15B-GA1 and *Tl*Ga15B-GA2 showed a significant increase of 8 °C (80 ℃) and 9 °C (81 ℃), respectively.

Remarkably, *Tl*Ga15B-GA1 and *Tl*Ga15B-GA2 also exhibited higher catalytic activity and efficiency than *Tl*Ga15B (Table 1; Additional file 6). The specific activities of *Tl*Ga15B-GA1 and *Tl*Ga15B-GA2 were approximately 1.6-fold and 2.2-fold higher than that of *Tl*Ga15B, respectively. In contrast to *Tl*Ga15B, the catalytic efficiencies of *Tl*Ga15B-GA1 and *Tl*Ga15B-GA2 increased by 55.7% and 305.4%, respectively. Usually, thermostability improvement of enzymes is accompanied by a decrease in catalytic activity due to the activity-stability trade-off [17, 18]. However, these results indicate that the activity-stability trade-off could be overcome by carefully selecting mutation sites distant from the catalytic center.

### MD simulation and DCCM analysis of *Tl*Ga15B and mutants

**Fig. 4:**
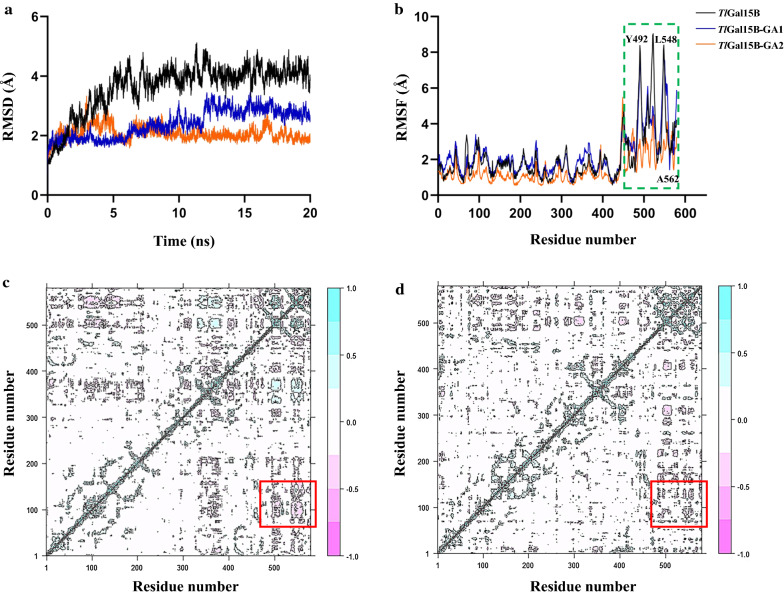
MD simulation analysis of *Tl*Ga15B and mutants *Tl*Ga15B-GA1 and *Tl*Ga15B-GA2. Comparison of RMSD values between WT and mutants calculated from 20-ns MD simulations at 300 K (**a**). Comparison of normalized RMSF between WT and mutants (**b**). DCCM analysis of *Tl*Ga15B (**c**) and *Tl*Ga15B-GA2 (**d**) based on the MD simulation trajectories

In order to elucidate the mechanism underlying the thermostability of mutants *Tl*Ga15B-GA1 and *Tl*Ga15B-GA2, MD simulations were carried out at 300 K for 20 ns. The root mean square deviation (RMSD) was an important parameter to reflect the rigidity of the protein structure, which correlated with protein thermostability [35]. The RMSD values of *Tl*Ga15B-GA1 and *Tl*Ga15B-GA2 significantly decreased compared with that of *Tl*Ga15B, indicating that the overall structures of the mutants were more thermostable (Fig. 4a). Moreover, based on the root mean square fluctuation (RMSF) analysis, residues Q108, S132, Y492, L548, and A562 of *Tl*Ga15B were more flexible than the corresponding ones of the mutants *Tl*Ga15B-GA1 and *Tl*Ga15B-GA2 (Fig. 4b). Besides, the flexibilities of amino acid residues in the CBM domain of thermostable mutants were markedly decreased. The lower RMSF values of these amino acid residues rendered the CBM domain more stable, which might result in improved catalytic efficiency of *Tl*Ga15B-GA1 and *Tl*Ga15B-GA2.

In addition, to understand the complex interaction between the selected mutated amino acid residues and the CBM domain, the DCCMs of *Tl*Ga15B and *Tl*Ga15B-GA2 were calculated using the coordinates of the Cα atoms from the trajectories. As shown in Fig. 4c, d, the negative correlation between the amino acid residues (100–120) and the CBM domain (residues 490–613) was weakened in the mutant *Tl*Ga15B-GA2, thereby indicating a marked reduction in the movement of the negative correlation between them. These results were consistent with the conclusions of the RMSF analysis.

### Starch saccharification using *Tl*Ga15B-GA2 in combination with pullulanase

**Fig. 5:**
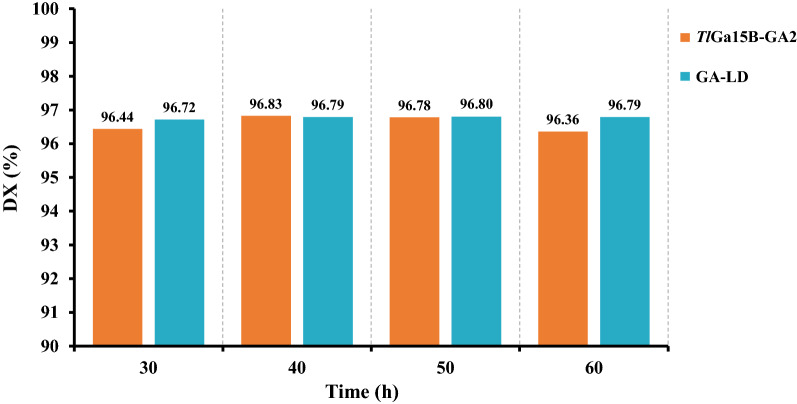
Comparison of the starch saccharification effect between *Tl*Ga15B-GA2 and the commercial glucoamylase GA-LD in glucose production at 60 °C

In order to evaluate the performance of industrial saccharification applications, the best mutant *Tl*Ga15B-GA2 was chosen for enzymatic saccharification of liquefied starch. As shown in Fig. 5, the maximum glucose productions for *Tl*Ga15B-GA2 and the commercial glucoamylase GA-LD were obtained after 30 h of incubation at 60 °C. The DX value (glucose content) for *Tl*Ga15B-GA2 and GA-LD reached 96.4% and 96.7%, respectively, indicating no significant difference between *Tl*Ga15B-GA2 and the commercial glucoamylase GA-LD. Moreover, the DX value of *Tl*Ga15B-GA2 was in accordance with the industrial requirement for the production of glucose with a DX value above 96%. These results suggested that *Tl*Ga15B-GA2 had the same saccharification effect as industrial glucoamylases for the enzymatic saccharification process of starch.

## Conclusions

*Tl*Ga15B, derived from thermophilic fungus *T. leycettanus* JCM12802, exhibited high optimal temperature and excellent thermostability above 60 ℃. By introducing disulfide bonds and optimizing residual charge–charge interactions in a region distant from the catalytic center, *Tl*Ga15B-GA1 and *Tl*Ga15B-GA2 showed improvements in optimal temperature, melting temperature, specific activity, and catalytic efficiency. The mechanism underlying the improved thermostability and catalytic efficiency of glucoamylase was elucidated by MD simulation and DCCM analysis. The performance of *Tl*Ga15B-GA2 was similar to that of the commercial glucoamylase during the saccharification process of starch. These properties of *Tl*Ga15B-GA2 make it a promising candidate for industrial saccharification applications.

## Methods

### Strain and substrates

The thermophilic fungus *T. leycettanus* JCM12802 was purchased from the Japan Collection of Microorganisms RIKEN BioResource Center (Tsukuba, Japan) and was maintained at 4 ℃ on a potato dextrose agar plate. Soluble starch, amylose, glycogen, α-cyclodextrin, β-cyclodextrin, and γ-cyclodextrin were purchased from Sigma-Aldrich. All other chemicals were of analytical grade and commercially available.

### Cloning of *Tl*Ga15B and mutants

*T. leycettanus* JCM12802 was cultured in the potato dextrose broth (PDB) medium at 40 ℃ for 3 days. Total RNA was extracted using the TRIZOL reagent (Invitrogen, Waltham, MA, USA) according to the operation manual. The first strand cDNA was synthesized from the total RNA using the FastKing RT Kit with gDNase (Tiangen, Beijing). Based on the 5′ and 3′-end sequences of the *Tl*Ga15B structural gene, the *Tl*Ga15B-encoding gene devoid of the signal peptide sequence was amplified with gene-specific primers (as shown in Additional file 7). The PCR product was cloned into the plasmid pPIC9 to construct a recombinant plasmid pPIC9-*Tl*Ga15B. Site-directed mutants pPIC9-*Tl*Ga15B-GA1 and pPIC9-*Tl*Ga15B-GA2 were constructed using the Fast Mutagenesis System Kit (TransGen, Beijing) with the plasmid pPIC9-*Tl*Ga15B as the template.

### Expression and purification of *Tl*Ga15B and mutants

The recombinant plasmid PIC9-*Tl*Ga15B, pPIC9-*Tl*Ga15B-GA1, and pPIC9-*Tl*Ga15B-GA2 were linearized with *Bgl*II and transformed into *P. pastoris* GS115 competent cells, respectively. The positive transformant with the highest glucoamylase activity was inoculated into YPD medium at 30 °C for 2 days with shaking at 200 rpm and used as the inoculum of 300 mL BMGY medium. The cultures were grown at 30 °C for 2 days, and cells were then harvested and resuspended in 200 mL BMMY medium for 2 days growth at 30 °C. The crude enzyme was collected by centrifugation at 12,000*g* for 10 min at 4 °C, followed by concentration through a 10 kDa cut-off centrifuge filter. The crude enzyme was dialyzed in citrate–phosphate buffer (20 mM, pH 6.3) at 4 °C overnight using a 3 kDa dialysis tube (Vivascience, Hannova, Germany), and run through a HiTrapTM Q Sepharose XL anion exchange column (GE Healthcare, Munich, Germany) for purification. The purified recombinant *Tl*Ga15B and mutants were verified by SDS-PAGE using a 10% polyacrylamide gel.

### Glucoamylase activity assay

The glucoamylase activity of *Tl*Ga15B and mutants was determined using soluble starch as the substrate. Reactions containing 900 μL of 1% soluble starch in citrate–phosphate buffer (pH 4.5) and 100 μL of appropriately diluted enzyme solution were incubated at 60 °C for 30 min, using the 3,5-dinitrosalicylic acid (DNS) method to detect the amount of reducing sugar in the reaction. One unit (U) of enzyme activity was defined as the amount of enzyme that released 1 μmol of glucose per minute.

### Biochemical characterization of purified *Tl*Ga15B and mutants

The effects of pH on the activity of purified *Tl*Ga15B and mutants were determined in glycine–HCl (pH 1–3), citric acid-Na_2_HPO_4_ (pH 3–8), and glycine–NaOH (pH 8–12) buffers at 37 ℃. To determine pH stability, the purified *Tl*Ga15B and mutants were incubated in different pH (1–12) for 1 h at 37 ℃. The effect of temperature on activity was measured in citric acid-Na_2_HPO_4_ buffer (pH 4.5) at 20–90 ℃. To evaluate thermostability, the purified *Tl*Ga15B and mutants were incubated at 75 ℃ for different time periods (0–60 min).

Kinetic studies were performed in a citric acid-Na_2_HPO_4_ buffer (pH 4.5) at 55 ℃ using 1.0–10.0 mg/mL soluble starch as the substrates. The Lineweaver–Burk plot method was used to calculate the *K*_m_ and *V*_max_ values of the purified *Tl*Ga15B and mutants. The substrate specificities of *Tl*Ga15B were studied in terms of the hydrolysis of six different substrates, including soluble starch, amylose, glycogen, α-cyclodextrin, β-cyclodextrin, and γ-cyclodextrin in a citric acid-Na_2_HPO_4_ buffer (pH 4.5).

### Differential scanning calorimetry (DSC) analysis

The melting temperatures (*T*_m_) of *Tl*Ga15B and mutants were determined using MicroCal™ VP-Capillary DSC (GE Healthcare). The purified *Tl*Ga15B and mutants were dissolved in a 10 mM phosphate buffer (pH 7.4) as an infusion, and the protein concentration was controlled at 0.50 mg/mL. The temperature program was run in the range of 50–100 ℃, with a rising rate of 1 ℃/min and a scanning rate of 1℃/min.

### Molecular dynamics (MD) simulation and dynamics cross-correlation matrix (DCCM) analysis

MD simulations were performed using the AMBER14 package at a temperature of 300 K for 20 ns with ff99SB force field. The closest distance between periodic box and atom was set 12 Å, and the time step was set to 2 fs. Before MD simulation, hydrogen atoms were added and any water molecules that did not interact with the protein were removed, and 20 mM sodium chloride was added to neutralize the charge in the system. The conjugate gradient method with α-carbon atoms restriction was used for energy minimization. The energy was minimized again without limiting the atoms, and finally the temperature was raised from 0 to 300 K. The MD trajectory data were analyzed using the CPPTRAJ software [36]. In addition, the last 10 ns trajectories from MD simulations were used to perform DCCM analysis using the Bio3D packages [37, 38].

### Application of recombinant *Tl*Ga15B-GA2 in starch saccharification

To evaluate the industrial application potential of *Tl*Ga15B-GA2 in starch saccharification, *Tl*Ga15B-GA2 was compared with the commercial glucoamylase GA-LD from Shandong Longda Bio-products Co Ltd. The saccharification process of starch was carried out at 60 °C for 30, 40, 50, and 60 h, containing 50 U/g *Tl*Ga15B or GA-LD, 0.6 U/g pullulanase, and 200 g of liquefied starch. The enzymatic saccharification effect of starch was measured by monitoring the DX value (glucose content) of the saccharification solution.

## Supplementary Information

**Additional file 1**: The Ramachandran plot of homology modeling for *Tl*Ga15B.

**Additional file 2**: SDS-PAGE analysis of the recombinant *Tl*Ga15B and mutants. Lane 1, 3, 5, the culture supernatant of transformants *Tl*Ga15B, *Tl*Ga15-GA1, and *Tl*Ga15-GA2; lane 2, 4, 6, the purified *Tl*Ga15B, *Tl*Ga15-GA1, and *Tl*Ga15-GA2.

**Additional file 3**: The thermostability of the purified recombinant *Tl*Ga15B.

**Additional file 4** LC-MS/MS analysis of the formation of disulfide bonds in the *Tl*Ga15B mutants. a: the disulfide-linked peptide SNPSGGLCT/SASGPCA (132C/492C); b: the disulfide-linked peptide PLWYCIV/SAIPCSA (548C/562C).

**Additional file 5**: The modeled structure of *Tl*Ga15B-GA2. Six candidate mutated sites away from the catalytic center were indicated in blue balls. Two disulfide bonds were indicated as yellow sticks.

**Additional file 6**: The specific activity of *Tl*Ga15B and its mutants in the temperature range from 20 to 90 ℃.

**Additional file 7**: Primers used in this study.

## Data Availability

The datasets used and/or analyzed during the current study are available from the corresponding author on reasonable request.

## Abbreviations

ETSS: Enzyme thermal stability system
RMSD: Root mean square deviation
RMSF: Root mean square fluctuation
PDB: Potato dextrose broth
DNS: 3,5-Dinitrosalicylic acid
MD: Molecular dynamics
DCCM: Dynamics cross-correlation matrix
