# Supplementary figures and images for: Improvement of thermostability and catalytic efficiency of glucoamylase from Talaromyces leycettanus JCM12802 via site-directed mutagenesis to enhance industrial saccharification applications
Source: Biotechnol Biofuels. 2021 Oct 16;14:202. doi: 10.1186/s13068-021-02052-3 (PMC8520190; doi:10.1186/s13068-021-02052-3)

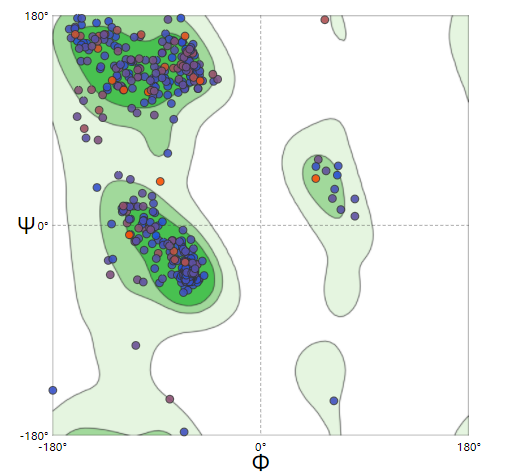
**Additional file 1.** The Ramachandran plot of homology modeling for *Tl*Ga15B.

Supplement: Supplementary file 1 — Additional file 1: The Ramachandran plot of homology modeling for TlGa15B. [file 13068_2021_2052_MOESM1_ESM.docx]
