# Supplementary material for: Improvement of thermostability and catalytic efficiency of glucoamylase from Talaromyces leycettanus JCM12802 via site-directed mutagenesis to enhance industrial saccharification applications
Source: Biotechnol Biofuels. 2021 Oct 16;14:202. doi: 10.1186/s13068-021-02052-3 (PMC8520190; doi:10.1186/s13068-021-02052-3)

**Additional file 2.** SDS-PAGE analysis of the recombinant *Tl*Ga15B and mutants. Lane 1, 3, 5, the culture supernatant of transformants *Tl*Ga15B, *Tl*Ga15-GA1 and *Tl*Ga15-GA2; lane 2, 4, 6, the purified *Tl*Ga15B, *Tl*Ga15-GA1 and *Tl*Ga15-GA2.


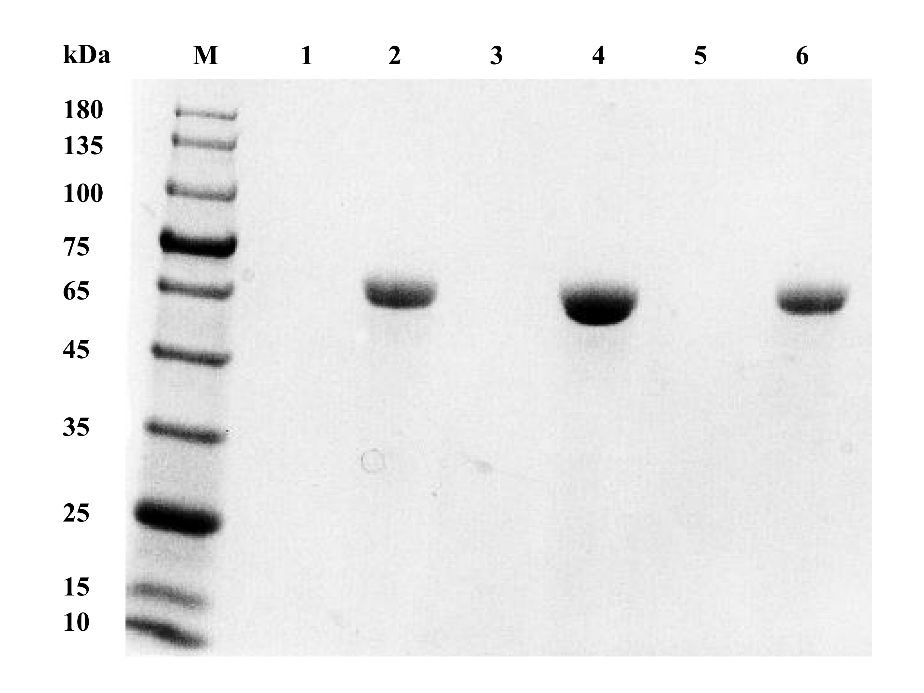

Supplement: Supplementary file 2 — Additional file 2: SDS-PAGE analysis of the recombinant TlGa15B and mutants. Lane 1, 3, 5, the culture supernatant of transformants TlGa15B, TlGa15-GA1, and TlGa15-GA2; lane 2, 4, 6, the purified TlGa15B, TlGa15-GA1, and TlGa15-GA2. [file 13068_2021_2052_MOESM2_ESM.docx]
