# Supplementary material for: Improvement of thermostability and catalytic efficiency of glucoamylase from Talaromyces leycettanus JCM12802 via site-directed mutagenesis to enhance industrial saccharification applications
Source: Biotechnol Biofuels. 2021 Oct 16;14:202. doi: 10.1186/s13068-021-02052-3 (PMC8520190; doi:10.1186/s13068-021-02052-3)

**Additional file 3.** The thermostability of the purified recombinant *Tl*Ga15B in the temperature range from 55 to 75 ℃.


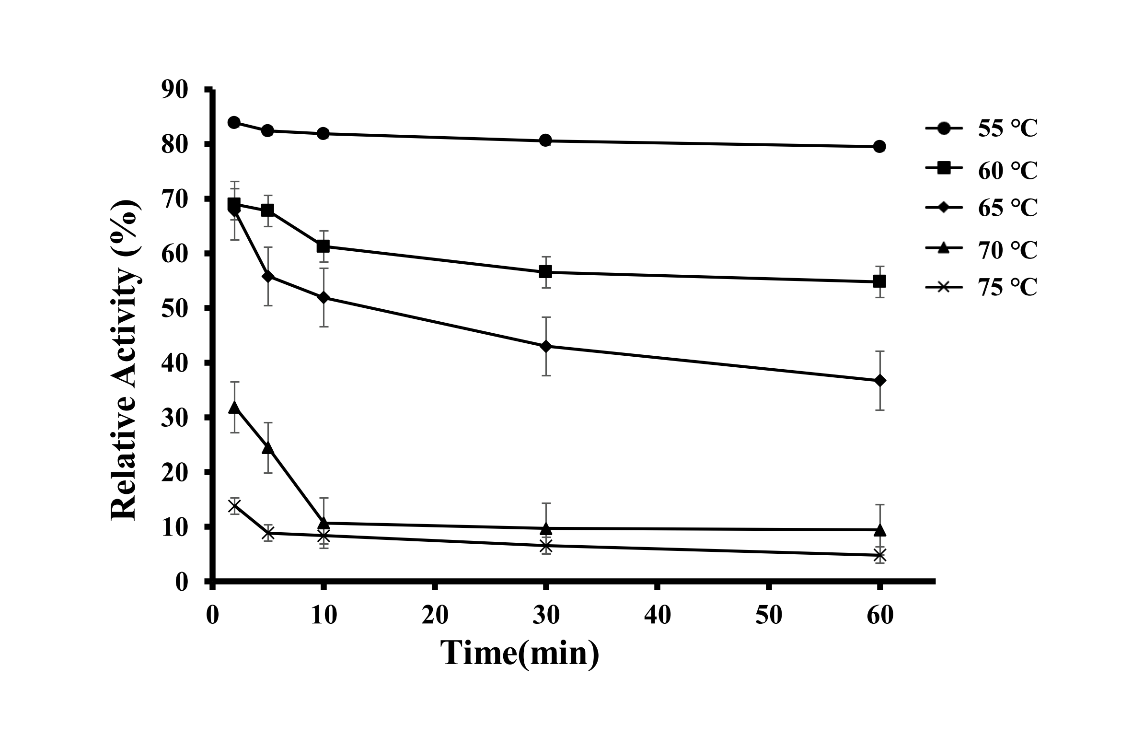

Supplement: Supplementary file 3 — Additional file 3: The thermostability of the purified recombinant TlGa15B. [file 13068_2021_2052_MOESM3_ESM.docx]
