# Supplementary material for: Improvement of thermostability and catalytic efficiency of glucoamylase from Talaromyces leycettanus JCM12802 via site-directed mutagenesis to enhance industrial saccharification applications
Source: Biotechnol Biofuels. 2021 Oct 16;14:202. doi: 10.1186/s13068-021-02052-3 (PMC8520190; doi:10.1186/s13068-021-02052-3)
Supplement: Supplementary file 4 — Additional file 4 LC-MS/MS analysis of the formation of disulfide bonds in the TlGa15B mutants. a: the disulfide-linked peptide SNPSGGLCT/SASGPCA (132C/492C); b: the disulfide-linked peptide PLWYCIV/SAIPCSA (548C/562C). [file 13068_2021_2052_MOESM4_ESM.docx]

**
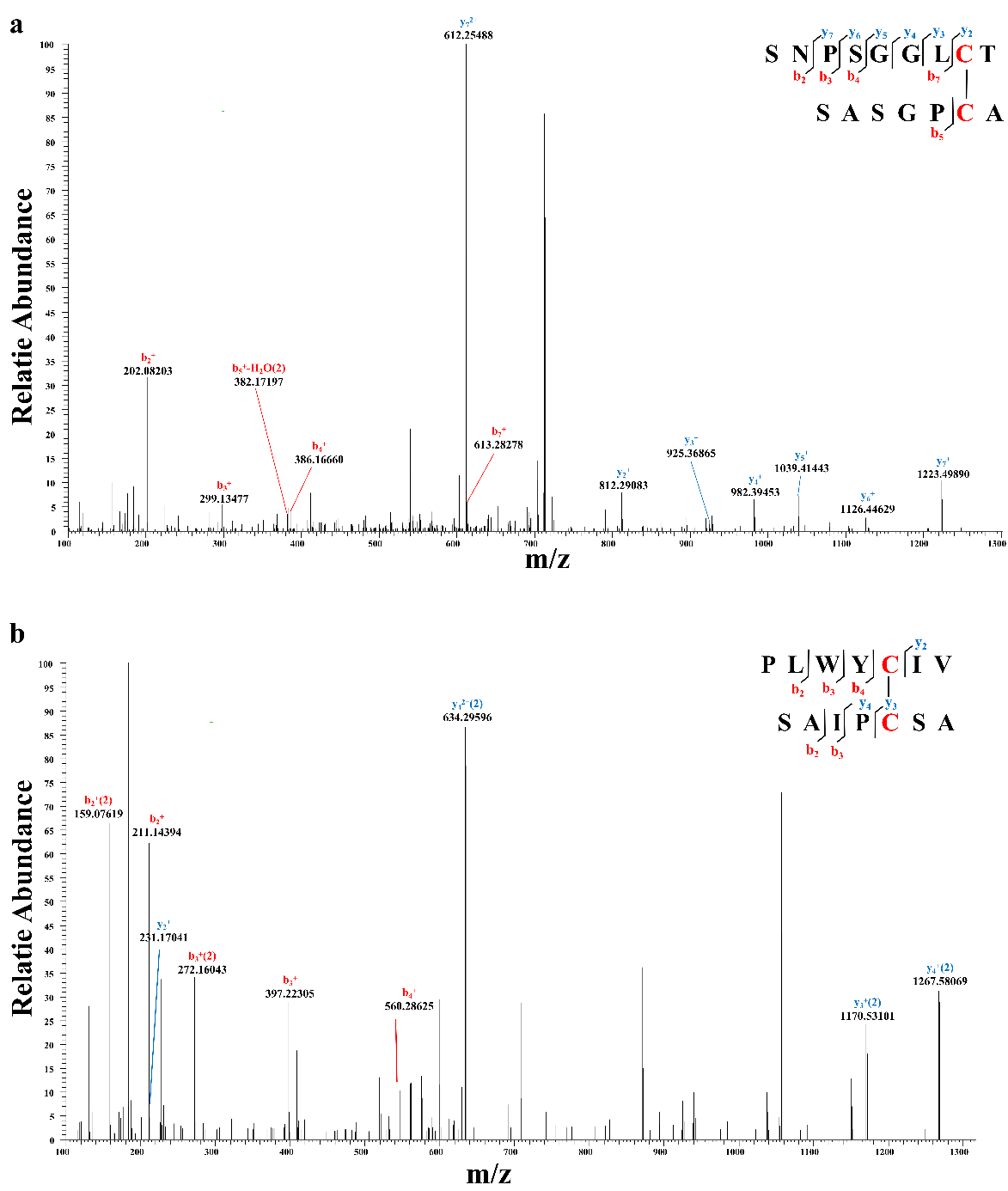
Additional file 4.** LC-MS/MS analysis of the formation of disulfide bonds in the *Tl*Ga15B mutants. a: the disulfide-linked peptide SNPSGGLCT/SASGPCA (132C/492C); b: the disulfide-linked peptide PLWYCIV/SAIPCSA (548C/562C).
