# Supplementary material for: Improvement of thermostability and catalytic efficiency of glucoamylase from Talaromyces leycettanus JCM12802 via site-directed mutagenesis to enhance industrial saccharification applications
Source: Biotechnol Biofuels. 2021 Oct 16;14:202. doi: 10.1186/s13068-021-02052-3 (PMC8520190; doi:10.1186/s13068-021-02052-3)
Supplement: Supplementary file 5 — Additional file 5: The modeled structure of TlGa15B-GA2. Six candidate mutated sites away from the catalytic center were indicated in blue balls. Two disulfide bonds were indicated as yellow sticks. [file 13068_2021_2052_MOESM5_ESM.docx]

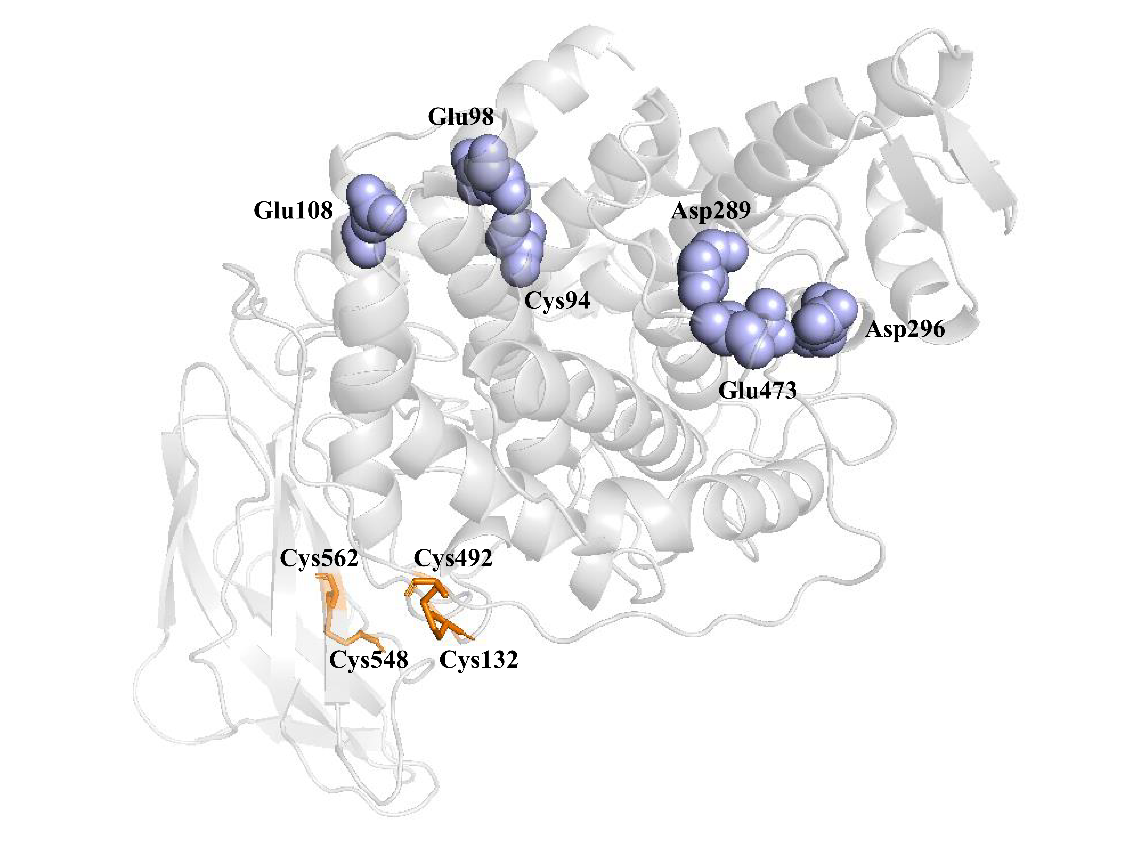
**Additional file 5.** The modeled structure of *Tl*Ga15B-GA2. Six candidate mutated sites away from the catalytic center were indicated in blue balls. Two disulfide bonds were indicated as yellow sticks.
