# Supplementary material for: Improvement of thermostability and catalytic efficiency of glucoamylase from Talaromyces leycettanus JCM12802 via site-directed mutagenesis to enhance industrial saccharification applications
Source: Biotechnol Biofuels. 2021 Oct 16;14:202. doi: 10.1186/s13068-021-02052-3 (PMC8520190; doi:10.1186/s13068-021-02052-3)
Supplement: Supplementary file 6 — Additional file 6: The specific activity of TlGa15B and its mutants in the temperature range from 20 to 90 ℃. [file 13068_2021_2052_MOESM6_ESM.docx]

**
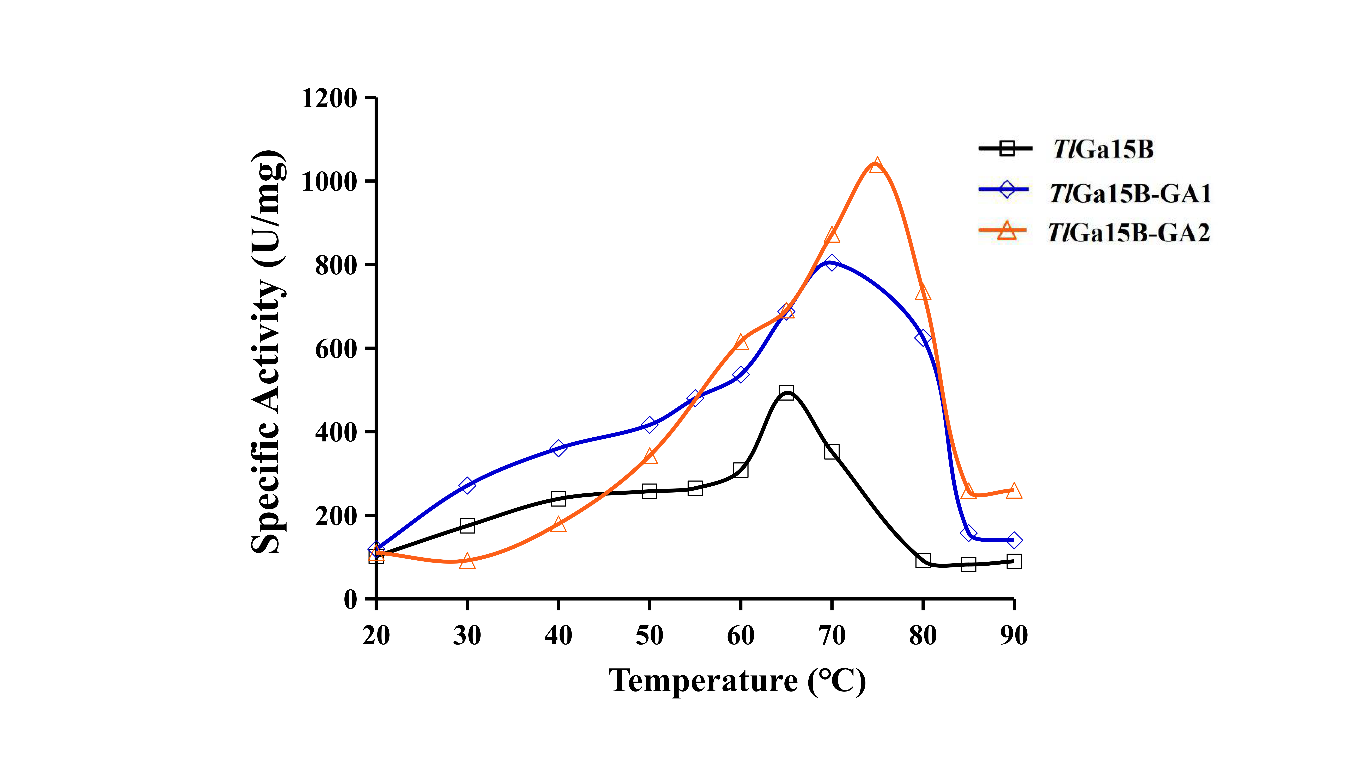
Additional file 6.** The specific activity of *Tl*Ga15B and its mutants in the temperature range from 20 to 90 ℃.
