# Supplementary material for: Improvement of thermostability and catalytic efficiency of glucoamylase from Talaromyces leycettanus JCM12802 via site-directed mutagenesis to enhance industrial saccharification applications
Source: Biotechnol Biofuels. 2021 Oct 16;14:202. doi: 10.1186/s13068-021-02052-3 (PMC8520190; doi:10.1186/s13068-021-02052-3)
Supplement: Supplementary file 7 — Additional file 7: Primers used in this study. [file 13068_2021_2052_MOESM7_ESM.docx]

**Additional file 7.** Primers used in this study.

| **Primers** | **Sequences (5'→3')*** |
| --- | --- |
| *Tl*Ga15B-F | TGAAGCTTACGTAGAATTCGCACCACATCCCACGGAACTT |
| *Tl*Ga15B-R | TAAGGCGAATTAATTCGCGGCCGCCTACCTCCAACTATCATT |
| S132C-F | CTCTGGCGGCCTGTGTACTGGAGGTC |
| S132C-R | ACACAGGCCGCCAGAGGGGTTGGACAC |
| Y492C-F | CTGCCTCAGGCCCCTGTGCCACCGCGAC |
| Y492C-R | ACAGGGGCCTGAGGCAGACGAGGCCGA |
| L548C-F | AGTTCCGCTATCCCGTGTAGCGCGGCCGA |
| L548C-R | ACACGGGATAGCGGAACTAGGACTCCAA |
| A562C-F | CGCCGTTGTGGTATTGTATCGTGACGTT |
| A562C-R | ACAATACCACAACGGCGTCTGTGAGTT |
| Q108E-F | TGGGGATGCGAACCTGGAGTCGGTGAT |
| Q108E-R | TCCAGGTTCGCATCCCCAGTCGAGAT |

*The restriction sites were underlined.
